# Prognostic Factors in Recurrent Congenital Muscular Torticollis

**DOI:** 10.5704/MOJ.2103.007

**Published:** 2021-03

**Authors:** C Chotigavanichaya, P Phongprapapan, J Wongcharoenwatana, P Eamsobhana, T Ariyawatkul, K Kaewpornsawan

**Affiliations:** Department of Orthopaedic Surgery, Faculty of Medicine Siriraj Hospital, Bangkok, Thailand

**Keywords:** congenital muscular torticollis, unipolar release, bipolar release, recurrence deformity

## Abstract

**Introduction:**

Congenital muscular torticollis (CMT), primarily resulting from unilateral shortening and fibrosis of the sternocleidomastoid muscle. One of the common surgical complications is recurrent deformity. However, the associations between unipolar or bipolar release, age of the patient, and the recurrence of the disease are unclear. Therefore, the purpose of this study was to evaluate the factors associated with recurrence after surgery.

**Materials and Methods:**

A retrospective review was performed in 47 patients who were diagnosed with CMT and had been treated surgically with unipolar or bipolar release between January 2007 and December 2015. Demographic data (sex, sides, surgical technique, age at time of surgery, period of follow-up, complications and recurrence) were recorded.

**Results:**

Forty-seven patients with an average age of 8.7 years old at time of surgery. Twenty-six patients had right-sided muscular torticollis, while 21 had left-sided. The average follow-up time was 2 years (range, 2–4 years). The average age of unipolar release was 8.8 years old (range, 218 years old), while the average age of bipolar release was 8.7 years old (range, 2–13 years old). Recurrence occurred in 11 patients (9 in unipolar and 2 in bipolar release). Sex, side of deformity, type of surgery and age at time of surgery showed no statistically significant as a factor for recurrence rate, however recurrence of unipolar more than bipolar surgery was nearly two times revealing clinical significance.

**Conclusions:**

Sex, side of deformity, type of surgery and age at time of surgery were not associated with the recurrence deformity.

## Introduction

Congenital muscular torticollis was the most common neck problem in children. The incidence was 1:300 live births^1^. Clinical evidence of torticollis presented initially as a palpable mass or tightness at the involved side; this may be a pseudotumor of the sternocleidomastoid muscle, which causes the cock robin posture^2^.

Etiology of this disease was still unknown^3^. However, previous literatures suggested causes are compartment syndrome during perinatal period from soft tissue compression of the neck at the time of delivery^4^; intrauterine crowding, based on the high association with breech presentation and developmental dysplasia of the hip^5^; primary neurogenic cause, histopathologic denervation and reinnervation due to a traumatic event^6^ (ischemia) and develop fibrosis of the sternocleidomastoid muscle; and the mesenchymal theory^7^, which is related to an environmental change, resulting in mesenchymal cells de-differentiating.

Initial treatment started with non-surgical intervention. Physical therapy program including stretching exercises^8^ showed excellent result in 90% of cases, especially in younger patients. Surgical treatment was recommended for patients who had persistent torticollis after 1-4 years of age^9^. Choices for surgical treatment were unipolar release or bipolar release. Recurrence was one of common complications after surgical release for torticollis. Shim JS *et al* reported proper time of surgery was the most important factor influences due to more prognosis was the proper time of surgery, that mean the patients can cooperate in bracing and rehabilitation program^10^. Chin En Chen and Jih Yang Ko found recurrence in 1 out of 18 torticollis patients^11^. The influences of sex, sides, surgical technique, and early versus delayed surgical release, on the torticollis recurrence rate remained unclear^12,13^. The purpose of this study was to evaluate the factors associated with recurrence of congenital muscular torticollis after surgery.

## Material and Method

A retrospective review was conducted on 47 congenital muscular torticollis patients who had tightness of the sternocleidomastoid muscle without an apparent mass. Aged between 2–15 years old. All cases were undergone surgical release between January 2007 and December 2015 and were followed-up at two, four and six weeks post-operative then every three months for two years and after that every six months. All cases had at least two years follow-up. The postoperative protocol included soft cervical collar for three weeks then physical therapy for 3-4 weeks. Demographic data (sex, sides of deformity, surgical technique, the age at time of surgery, the follow-up period, complications and recurrence) were recorded. Patients with secondary causes of muscular torticollis such as vertebral anomalies, unilateral atlantooccipital fusion, Klippel-Feil syndrome and unilateral absence of sternocleidomastoid were excluded. Patients with incomplete medical data were also excluded. The definition for recurrence was prominent appearance of sternocleidomastoid muscle with head tilt and 50% decreased in range of motions compare with first 12 weeks after surgery. Cox regression analysis was used to compare any of the associated prognostic factors and disease recurrence.

## Results

Forty-seven patients were included in the study Table I and II. The average age at time of surgery was 8.7 years old. Twenty-six patients (55.3%) had right-sided muscular torticollis, and twenty-one (44.7%) exhibited it on the left. Thirty-four patients (72.3%) had unipolar release, while 13 (27.7%) had bipolar release. The average follow-up time was 33.4 months. Recurrence of the deformity was found in 11 (23.4%) out of 47 patients; the mean recurrence time was 52.5 months (median: 52.8 months).

**Table I:** Demographic data

Patients characteristics	Mean ± SD / n (%) (n=47)
Age (yrs)	8.7±3.7
	9(2-18)*
Gender	
Male	20(42.6%)
Female	27(57.4%)
Affected side	
Left	21(44.7%)
Right	26(55.3%)
Surgical procedure	
Unipolar	34(72.3%)
Bipolar	13(27.7%)
Recurrence	
Yes	11(23.4%)
No	36(76.6%)
Lee Score	
Poor	11(23.4%)
Fair	8(17.0%)
Good	16(34.0%)
Excellent	12(25.5%)
Follow-up time (months)	33.4±12.0
	31.4(6.1-70.1)*

* Median (Min-Max)

**Table II:** Clinical Outcome

Factors	Recurrence	Non-recurrence	Univariate	
	(n=11)	(n=36)	Hazard ratio	p value
Age at surgery (yrs.)	9.5±4.5	8.5±3.5	1.07 (0.89, 1.29)	0.467
Gender				0.249
Male (ref)	3(27.3%)	17(47.2%)	2.39	
Female	8(72.7%)	19(52.8%)	(0.54, 10.48)	
Affected side				0.157
Right (ref)	4(36.4%)	22(61.1%)	2.75	
Left	7(63.6%)	14(38.9%)	(0.68, 11.14)	
Surgical procedure				0.428
Unipolar	9 (81.8%)	25(69.4%)	1.98	
Bipolar (ref)	2 (18.2%)	11(30.6%)	(0.37, 10.72)	

**Fig. 1:**
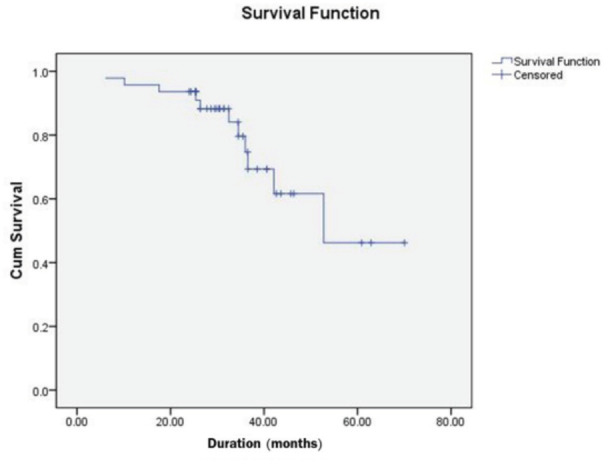
Survival curve for all congenital muscular torticollis patients. The first case of recurrence (1/11) was occurred at 6.1 months and last case of recurrence (11/11) was occurred at 52.8 months post-operative.

There was no statistically significant difference between recurrence of torticollis and the age at time of surgery (p=0.467), side of the torticollis (p=0.157) and gender (p=0.249). Moreover, there was no statistically significant difference between the risk of recurrence and technique of surgical release (p=0.428). From the Kaplan-Meier survival analysis (Fig. 1), first recurrence case developed at 6.1 months, and the patient with the longest follow-up time was a recurrence case developed in 52.8 months.

## Discussion

Congenital muscular torticollis was a common paediatric orthopedic problem. It may be associated with dysplasia of the hip or metatarsus adductus. Some theories suggested that intrauterine molding during pregnancy may cause this deformity^14^. Differential diagnoses of torticollis were basilar impression, C1-2 instability and neurologic torticollis.

The treatment of congenital muscular torticollis initially involved stretching exercises during the first year of age^8^. If patient’s neck remained tilted after one year of age, operative treatment was performed. Chandler^9^ advocated that good results can be achieved by performing the surgical procedure between one and four years of age.

In this study, there was nine cases (9/34) of recurrence after unipolar release and two cases (2/13) of recurrence after bipolar release. Even though there was no statistically significant between unipolar and bipolar release on recurrence rate, there was clinically significant difference when compared percentage of recurrence between two techniques (26.5% in unipolar release and 15.4% in bipolar release).

Previous literatures showed no recurrence case in patient presenting with CMT after 10 years old who were treated with bipolar release^15^. However, there was still controversy about which type of surgical treatment, unipolar or bipolar, provided better result. Chen *et al*^11^ reported a recurrence rate of 1 out of 18 patients (5.5%) from surgical treatment in congenital muscular torticollis patients aged over six years old. As for the long term results after open surgical tenotomy of the sternal and clavicular origins of the sternocleidomastoid muscle for idiopathic muscular torticollis, Ippolito *et al*^13^ reported that the factors affecting the treatment results were patient’s age at operation, the disease duration, and severity of the deformity before surgery.

Chen *et al*^11^ and Minamitani *et al*^16^ reported that excellent results in patients over six years of age and the degree of correction were affected by the adequacy of the surgical treatment and rehabilitation, rather than age. In this study, we evaluated the factors that may affect the post-operative recurrence of torticollis. However, no statistically significant difference was found between the recurrence of the deformity and sex, sides, age at time of surgical treatment and the type of surgery.

However, numerous studies have advocated that age at the of surgical treatment was a risk factor. For instance, it has been recommended that the ideal age for surgery should be between one and four years old^9^. Coventry *et al*^17^ suggested that good results can still be achieved at an upper age limit of 12–14 years. Lee *et al*^18^ suggested that patients between 6 and 16 years had good operative results, and concluded that the patient’s age at surgery seemed to be the most important factor. In contrast, Shim and Jang^10^ suggested that age was not the most important factor when determining the optimal time for the operation, but compliance with a post-operative rehabilitation program was the most important consideration. They recommended that congenital muscular torticollis surgery should be delayed until the patient’s compliance with the planned rehabilitation can be guaranteed.

In this study, recurrences occurred in 11 patients (23.4%), but they were not related to the age at time of surgery or the surgery type. The recurrences might occur due to the retained fibrous band or fascia of the sternocleidomastoid. As patients grew older, their body weight and height increase, thereby affecting the sternocleidomastoid fascia and vertebral bodies. This can lead to tightness of the fibrous band and resulting in a recurrence of torticollis.

The first case of recurrences developed early at 6.1 months after surgery, while the second case occurred in 10.1 months This suggested that the first follow-up appointment for clinical assessment should be scheduled at least 6 months after surgery, and subsequent one every 6–12 months for at least 7 years, to ensure that no recurrence has developed.

The limitations of this study were the small number of patients and the different surgeons may have employed varying decision-making techniques. We were also unable to evaluate the post-operative protocol due to the study’s retrospective nature.

## Conclusion

It is controversial whether unipolar or bipolar release produces better results. Sex, sides of deformity, type of surgery and age at the time of surgery were not associated with the recurrence of congenital muscular torticollis. Consequently, choices of surgery and the rehabilitation protocols should be discussed comprehensively, and the possibility of recurrence after surgical release should be advised.
